# Correction: Synthetic ramoplanin analogues are accessible by effective incorporation of arylglycines in solid-phase peptide synthesis

**DOI:** 10.1039/d3sc90245e

**Published:** 2024-01-04

**Authors:** Edward Marschall, Rachel W. Cass, Komal M. Prasad, James D. Swarbrick, Alasdair I. McKay, Jennifer A. E. Payne, Max J. Cryle, Julien Tailhades

**Affiliations:** a Department of Biochemistry and Molecular Biology, The Monash Biomedicine Discovery Institute, Monash University Clayton VIC 3800 Australia max.cryle@monash.edu julien.tailhades@monash.edu; b EMBL Australia, Monash University Clayton VIC 3800 Australia; c ARC Centre of Excellence for Innovations in Peptide and Protein Science Clayton VIC 3800 Australia; d Department of Microbiology, Monash University Clayton VIC 3800 Australia; e Department of Chemistry, Monash University Clayton VIC 3800 Australia

## Abstract

Correction for ‘Synthetic ramoplanin analogues are accessible by effective incorporation of arylglycines in solid-phase peptide synthesis’ by Edward Marschall *et al.*, *Chem. Sci.*, 2024, **15**, 195–203, https://doi.org/10.1039/D3SC01944F.

The authors regret that the number of distance restraints used to obtain the final structure ensemble was incorrectly stated on the sixth page of the manuscript. The corrected sentence should read: “The final structure ensemble was calculated in CYANA^67^ and is based on 330 assigned NOEs (Table S3 and Fig. S6†) which included 104 long-range NOEs and had good precision (backbone RMSD = 0.17 Å, heavy atom RMSD = 0.49 Å) and residual restraint violations.”

The Royal Society of Chemistry apologises for these errors and any consequent inconvenience to authors and readers.

## Supplementary Material

